# In Patients with Chronic Kidney Disease Advanced Glycation End-Products Receptors Isoforms (sRAGE and esRAGE) Are Associated with Malnutrition

**DOI:** 10.3390/antiox11071253

**Published:** 2022-06-25

**Authors:** Lara Caldiroli, Paolo Molinari, Elena Dozio, Roberta Rigolini, Paola Giubbilini, Massimiliano M. Corsi Romanelli, Giuseppe Castellano, Simone Vettoretti

**Affiliations:** 1 Unit of Nephrology, Dialysis and Kidney Transplantation, Fondazione IRCCS Ca’ Granda Ospedale Maggiore Policlinico di Milano, 20122 Milan, Italy; lara.caldiroli@policlinico.mi.it (L.C.); paolo.molinari1@unimi.it (P.M.); giuseppe.castellano@unimi.it (G.C.); 2 Laboratory of Clinical Pathology, Department of Biomedical Science for Health, Università degli Studi di Milano, 20133 Milan, Italy; elena.dozio@unimi.it (E.D.); mmcorsi@unimi.it (M.M.C.R.); 3 Service of Laboratory Medicine1-Clinical Pathology, IRCCS Policlinico San Donato, San Donato Milanese, 20097 Milan, Italy; roberta.rigolini@grupposandonato.it (R.R.); paola.giubbilini@grupposandonato.it (P.G.); 4 Department of Clinical Sciences and Community Health, Università degli Studi di Milano, 20122 Milan, Italy

**Keywords:** chronic kidney disease (CKD), malnutrition, advanced glycation end-products (AGEs), soluble receptor for AGE (sRAGE), cleaved RAGE (cRAGE), endogenous secretory RAGE (esRAGE), inflammation, carbonyl stress

## Abstract

Background: in patients with chronic kidney disease (CKD), the inflammatory and pro-oxidant milieu may contribute to malnutrition development. In this study, we investigated the relationship between inflammation, advanced glycation end-products (AGEs), and their receptors (RAGEs) with malnutrition in CKD patients. Methods: we evaluated 117 patients. AGEs were quantified by fluorescence intensity using a fluorescence spectrophotometer, soluble RAGEs isoforms, and inflammatory interleukins by ELISA. Malnutrition was assessed by a malnutrition inflammation score. Results: mean age was 80 ± +11 years, eGFR was 25 ± +11 mL/min/1.73 m^2^ and BMI was 28 ± 5 Kg/m^2^. Malnourished individuals were older, had lower estimated protein intake (nPCR 0.65 ± 0.2 vs. 0.8 ± 0.2 vs. 0.8 ± 0.3, *p* = 0.01), higher C reactive protein (CRP 0.6 ± 1 vs. 0.6 ± 0.7 vs. 0.17 ± 0.13, *p* = 0.02) and tumor necrosis factor α (TNF α 14.7 ± 8.7 vs. 15.6 ± 8 vs. 11.8 ± 5.8, *p* = 0.029). Malnourished patients had higher sRAGE (2813 ± 1477 vs. 2158 ± 1236 vs. 2314 ± 1115, *p* = 0.035) and esRAGE (648 [408–1049] vs. 476 [355–680] vs. 545 [380–730] *p* = 0.033). In the multivariate analysis, only sRAGE maintained its association with malnutrition (*p* = 0.02) independently of aging and inflammation. Conclusions: in CKD patients, RAGEs isoforms, but not AGEs, are associated with malnutrition, irrespective of systemic inflammation, aging, and renal function.

## 1. Introduction

The etiology of malnutrition in chronic kidney disease is complex and multifactorial: reduced food intake because of anorexia, changes in taste, uremic gastritis, and the high number of medicines prescribed may all contribute to reducing energy and protein intake [1,2,3]. CKD patients are also characterized by increased oxidative stress and inflammation due to the accumulation of uremic toxins and compromised renal clearance of pro-inflammatory mediators. Therefore, this pro-oxidant and inflammatory milieu may contribute to the development of malnutrition-inflammation syndrome, characterized by the coexistence of persistent low-grade inflammation and protein–energy malnutrition [4,5]. Advanced glycation end-products (AGEs) derive from non-enzymatic modifications of amino groups of proteins or lipids by reducing sugars and their metabolites. In CKD patients, AGEs may accumulate because of a reduction of their renal clearance and an increase in their production due to an imbalance between pro-oxidant/antioxidant homeostasis [6,7,8]. Moreover, uremic patients showed elevated AGE levels because of the very low activity of the glyoxalase detoxication system on reactive carbonyl compounds (RCOs), the precursors of pentosidine. In fact, the deficiency of glyoxalase I (GLO-I) determined a decrease in AGEs’ detoxification and plays a crucial role in increasing AGE levels in these patients [9].

AGEs, throughout the interaction with their receptors (advanced glycation end-products receptors, RAGEs), contribute to increased systemic inflammation. The accumulation of AGEs can increase circulating monocytic cell levels [10] and AGEs/RAGEs interaction activates Nf-Kβ, which is one of the principal transcriptional factors implied in the production of both pro-inflammatory cytokines [11,12]. In particular, AGEs accumulation is associated with an increased synthesis of TNFα, IL-6, and IL-1β [13,14]. Therefore, in CKD patients, AGEs accumulation initiates a sort of a vicious circle. In fact, if, on one side, AGEs production is enhanced by the pro-inflammatory milieu, on the other one, their accumulation induces a pro-inflammatory response [15].

In end-stage renal disease patients’, levels of circulating AGEs have been associated with the prevalence of malnutrition [2,14]. This may depend on several roles played by AGEs that can induce involuntary weight loss, such as increased inflammation, enhanced protein catabolism, and energy expenditure [2,16]. Starting from this evidence, we hypothesize that the increase in AGEs levels that characterizes advanced stages of CKD might actively contribute to the pathogenesis of malnutrition in this population. However, the detailed pathophysiology of the relationship between AGEs and malnutrition in CKD remains to be clarified. Therefore, in this observational study, we explored the interplay between malnutrition, AGEs, RAGEs isoforms, and inflammation in patients affected by advanced CKD.

## 2. Materials and Methods

### 2.1. Patients and Study Design

We evaluated cross-sectionally 117 prevalent patients between 9/2016 and 3/2018 that were enrolled according to the following criteria: age ≥ 65 years, CKD stages 3a to 5 in conservative therapy, and with a relatively stable glomerular filtration rate (GFR) over the previous 6 months (with less than 2 mL/min/1.73/m^2^ of variation). GFR was estimated according to the CKD-EPI formula (eGFR). We excluded patients with cancer, advanced cirrhosis and/or ascites, severe heart failure (NYHA class III–IV), nephrotic syndrome, thyroid diseases, bowel inflammatory diseases, and inability to cooperate. We also excluded patients treated with immunosuppressive drugs or who had been hospitalized in the last three months. Twenty-four hours urinary collection and biochemical parameters were collected the day of the visit after overnight fasting of at least 12 h. The study was conducted according to the ICP Good Clinical Practices Guidelines, and it was approved by the Ethics Committee of our Institution (Milano 2- approval N. 347/2010). All patients signed informed consent.

### 2.2. sRAGE, esRAGE and cRAGE Quantification

Quantification of total isoforms of RAGEs was performed as previously indicated [17]. Briefly, sRAGE and esRAGE were dosed using two ELISA kit: R&D Systems kit (DY1145, Minneapolis, MN, USA) for sRAGE, while for esRAGE we used B-Bridged International kit (K1009–1, Santa Clara, CA, USA). The intra- and inter-assay coefficients of variation of esRAGE assay were 6.37 and 4.78–8.97%, respectively. cRAGE levels were obtained by subtracting esRAGE from total sRAGE. The AGE/sRAGE ratio was then obtained. The GloMax^®^-Multi Microplate Multimode Reader was used for photometric measurements (Promega, Milan, Italy).

### 2.3. AGE Quantification

AGEs levels were dosed as previously reported [18,19]. Briefly, we measure the fluorescence intensity at 414–445 nm after excitation at 365 nm with a fluorescence spectrophotometer (The GloMax^®^, Promega). Fluorescence intensity was expressed in arbitrary units (AU). AGEs were then normalized for total protein content.

### 2.4. Interleukins Quantification

Serum cytokines concentration were dosed in duplicate using the following ELISA kits according to the manufacturer’s instructions:

Quantikine ELISA Human CCL2/MCP-1 Immunoassay DCP00, Human TNF-alpha ELISA Kit (Thermo Fisher Scientific, Monza, Italy) and Quantikine HS ELISA Human IL-6 Immunoassay HS600B (R&D Systems, Space, Milano, Italy). Zero was included, as the last standard value, in each resulting curve. Results were validated by using Quantikine Immunoassay Control Group 1–4 or 10 (R&D Systems, Space). Absorbance readings were measured at 450 nm by spectrophotometer (Xenius Safas, Monaco).

### 2.5. Nutritional Status

Nutritional status was assessed with malnutrition inflammation score (MIS). MIS consists of ten domains, each of which with a score scale 0–3 according to the severity level [20]. A total score of 4–7 was considered indicative of risk of malnutrition, and a score ≥8 of malnutrition [21].

Protein intake was determined by normalized protein catabolic rate (nPCR) on 24 h urinary urea excretion [22].

Energy intake was assessed with dietary diaries that were compiled the two days preceding the visit. We calculated nutrient intake using the nutritional software Winfood (Medimatica Srl, Teramo Italy).

We also measure the following anthropometric measurements: body weight, height, body mass index (BMI, calculated according to Quetelet Index (kg/m^2^)).

### 2.6. Statistical Analysis

We expressed continuous variables as mean with standard deviation (SD), in parametric distributions, or median with interquartile range (IQR), in non-parametric data. Categorical variables were summarized as percentages. We performed Student’s t-test and ANOVA to compare parametric variables, while we performed, when appropriate, the Mann–Whitney “U” test or Kruskal–Wallis for the comparison of not parametric ones. Multivariate linear regression was used to test for the correlation between inflammatory interleukins, AGEs, and RAGEs isoforms; correcting for the influence of eGFR.CRP levels were considered as Log-values, in this case, to compensate for nonlinear distribution. We also evaluated the correlation of malnutrition, as a dependent variable, with sRAGE and esRAGE in two multivariable logistic regression models that included all the principal variables that were associated or had an interaction with AGEs and malnutrition.

Statistical Analysis was conducted using IBM SPSS software (version 26, IBM, Armonk, NY, USA).

## 3. Results

### 3.1. Population Characteristics

Our study enrolled 117 patients whose general characteristics are depicted in Table 1. The mean age was 80 ± 11 years. A total of 70% of patients were males. Nearly half of the patients had diabetes (56%), and, on average, they were overweight, with a mean BMI of 28 ± 5 kg/m^2^. Median malnutrition inflammation score was 4 [3–8], while eGFR ranged from 60 to 15 mL/min/1.73 m^2^, with a mean value of 25 ± 11 mL/min/1.73 m^2^ (stage IV CKD).

Nutritional metabolic markers were averagely normal (total cholesterol 168 ± 37 mg/dL; albumin 4.0 ± 0.4 gr/dL; prealbumin 28 ± 5 mg/dL).

Regarding inflammatory markers, CRP levels were generally increased in our cohort (0.4 ± 0.7 mg/dL). Mean TNFα, IL6 and MCP-1 levels were (15.3 ± 8.2 pg/m; 4.2 ± 3.1 pg/mL and 410 ± 176 pg/mL respectively).

### 3.2. General Cohort Characteristics, Inflammation, and Nutritional Status

As evident in Table 2, malnourished patients were older (82 ± 5 vs. 77 ± 8 and 72 ± 17 years respectively; *p* = 0.002) and had an individual protein intake (estimated by nPCR) that was lower than non-malnourished and at-risk for malnutrition patients (0.65 ± 0.2 vs. 0.8 ± 0.3 and 0.8 ± 0.2 gr/kg respectively; *p* = 0.01). Conversely, energy intake was comparable in the three groups (21 ± 6 vs. 22 ± 6 and 22 ± 9 Kcal/kg respectively, *p*= 0.8)

Concerning serum parameters, malnourished patients had lower concentrations of serum albumin (3.8 ± 0.2 vs. 4.1 ± 0.4 and 4.2 ± 0.3 gr/dL respectively; *p* = 0.002) and prealbumin (27 ± 6 vs. 27 ± 5 and 32 ± 5 mg/dL respectively; *p* < 0.0001). No difference concerning eGFR, lipid profile, and BMI was evident between the different groups.

Concerning inflammatory markers (Table 3), both CRP (0.6 ± 1 vs. 0.6 ± 0.7 and 0.2 ± 0.1 mg/dL respectively, *p* = 0.029) and TNFα (15 ± 9 vs. 16 ± 8 and 12 ± 6 pg/mL respectively; *p* = 0.029) levels were significantly higher in malnourished patients compared to the other groups.

### 3.3. AGEs and RAGEs Isoforms and Nutritional Status

**Figure 1:**
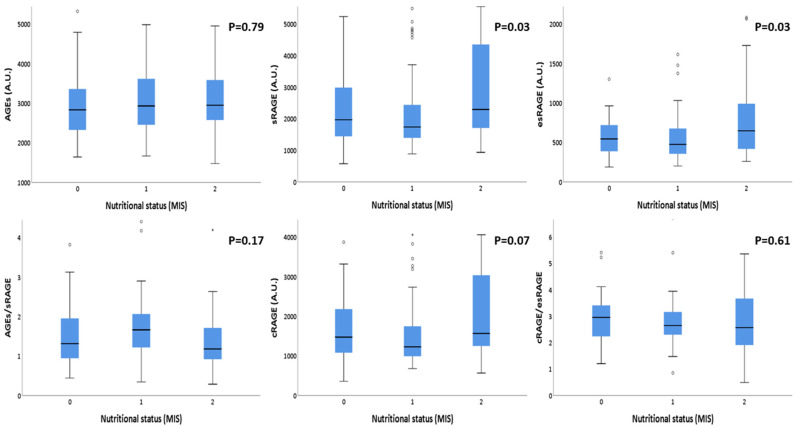
Box plot representation of AGEs, sRAGE isoforms’ distributions according to different nutritional status, evaluated by MIS. Note: AGEs: Advanced Glycation End products; sRAGE: soluble receptor for AGE; esRAGE: endogenous secretory receptor for AGE; cRAGE: cleaved receptor for AGE; MIS: malnutrition-inflammation score. Nutritional status has been grouped according to the severity: 0: non malnourished patients (MIS between 0 and 3); 1: patients at risk for malnutrition (MIS between 4 and 7); 2: malnourished patients (MIS equal or greater than 8).

The correlations between AGEs and RAGEs with the nutritional status are summarized in Table 4. Although AGEs concentrations did not show any significant correlation, RAGEs isoforms showed significant statistical differences according to nutritional status categorization. In particular: sRAGE and esRAGE were significantly higher in malnourished patients compared with those at risk of malnutrition and well-nourished ones *p* = 0.035 and *p* = 0.033, respectively). AGE/sRAGE ratio and cRAGE/esRAGE ratio did not show any statistical difference between different nutritional statuses. In Figure 1, these results are better summarized. In fact, in this box plot representation, the significant rise in sRAGE and esRAGE values with the worsening of the nutritional status is clearly evident. The same pattern, even if it did not reach statistical significance, was noticeable for cRAGE distribution.

We also performed additional analyses to test for eventual correlation between AGEs, sRAGE, and relative isoforms and the main biochemical parameters evaluated in our cohort. These results are shown in detail in Table S2 in Supplementary Materials. The main result was the strong negative correlation between all sRAGE isoforms (e.g., sRAGE, esRAGE, and cRAGE) and nPCR (*p* < 0.0001, *p* = 0.003, *p* = 0.001 respectively). Secondarily, lower AGEs/sRAGE values were significantly correlated with lower albumin and nPCR values (*p* = 0.005 and *p* = 0.01 respectively).

### 3.4. AGEs and RAGEs Isoforms Association with Inflammatory Markers

We also performed additional analyses to test for eventual correlation between AGEs, sRAGE, and relative isoforms and pro-inflammatory markers. First of all, AGEs levels were directly and significantly correlated with CRP levels (*p* = 0.05), while sRAGE and cRAGE were negatively correlated with it (*p* = 0.02 and *p* = 0.01, respectively). Secondarily, higher values of AGEs/sRAGE ratio significantly correlated with higher CRP values (*p* < 0.0001). We performed a series of multivariate linear regression analyses to evaluate whether there was any relationship between AGEs, RAGEs, and their ratio with inflammatory markers that were mainly associated with malnutrition (i.e., CRP and TNFα). Since CRP had a skewed distribution, it was log-transformed for the analysis. Moreover, considering that AGEs, RAGEs, CRP, and TNFα levels are influenced by eGFR, all models were corrected for patients’ eGFR (results of these analyses are reported in Figures S1 and S2 in Supplementary Materials). Although both sRAGE and AGEs/sRAGE levels were in general linked to an increase in pro-inflammatory markers, only the association of CRP with sRAGE and AGEs/sRAGE reached the statistical significance (B = 0.006, *p* = 0.049 and B = 0.294, *p* = 0.03 respectively).

### 3.5. AGEs and RAGEs Isoforms Association with Malnutrition Development

We finally designed two multivariate models to evaluate the eventual association of sRAGE and esRAGE with malnutrition. We built this model after performing interaction analyses, which are shown in Table S2 in Supplementary Materials. The main factors interacting with sRAGE for malnutrition development were Age, CRP and Sex (*p* = 0.003; *p* = 0.03 and *p* = 0.017, respectively). The same interactions were observed for esRAGE (Age, *p* = 0.002; CRP, *p* = 0.02; Sex, *p* = 0.004). Then, we created two different models, including separately sRAGE or esRAGE (in order to avoid redundancy in the model) with the principal variables that were associated or showed any interaction with overt malnutrition (Table 5).

sRAGE (Table 5) maintained its strength of association with overt malnutrition even after CRP, Sex and patients’ age were inserted into the model (OR 2.2; 95% CI 1.1–3.4; *p* = 0.035). However, CRP and age maintained the strongest association with malnutrition (OR 12.13; 95% CI 7.4–20.5; *p* = 0.001; OR 1.41; 95% CI 1.05–1.23, *p* = 0.001, respectively).

In Table 5, we built the same model replacing sRAGE with its spliced isoform, esRAGE. In this case, the association of esRAGE with malnutrition was non statistically significant, even if a clear trend of association was evident (OR 1.33; 95% CI 0.98–2.38; *p* = 0.055). Furthermore, in this case, CRP and age were the variables with the strongest association with malnutrition (OR 11.9 95% CI 6.8–20.6, *p* = 0.001; OR 1.41; 95% CI 1.05–1.23, *p* = 0.001, respectively).

## 4. Discussion

In our study, we confirmed that in patients with advanced CKD, chronic moderate inflammation is associated with malnutrition. Alongside that, we found that sRAGE concentration and AGEs/sRAGE ratio are associated with inflammatory markers, suggesting that AGEs/RAGEs interaction might modulate inflammation. Above all, we found that in CKD patients, sRAGE levels are associated with malnutrition independently of other well-known risk factors. Previous studies investigating the relationships between AGEs, sRAGE, and malnutrition in CKD were focused on end-stage renal disease patients undergoing chronic dialysis. To our knowledge, this is the first study that evaluated the correlation between inflammation, AGEs, RAGEs isoforms, and malnutrition in CKD patients not yet in dialysis.

Among patients with CKD, the coexistence of malnutrition and inflammation is a well-recognized condition [1,23,24,25]. In these patients, systemic inflammation begins to increase when the glomerular filtration rate drops under 60 mL/min, and its severity progresses with the decline of renal function. Accumulation of uremic toxins, imbalance in gut microbiota (translocation of endotoxins to systemic circulation), and the dysregulation of the immune system (macrophages exposed to uremic toxins) contribute to an increase in the production of TNFα and CRP [26]. The inflammatory milieu can affect nutritional status in several ways. TNFα promotes a catabolic state by inducing anorexia and engendering both protein degradation and suppression of their synthesis [27,28,29]. Therefore, uremic patients with a higher inflammatory state are prone to develop a negative protein balance because of a shift from anabolic protein synthesis to acute-phase protein production [29,30]. In a recent observational study conducted by Wang et al., higher MIS was associated with higher IL-6 levels [31]. Moreover, in a study by Keren et al., malnutrition, evaluated by MIS, was associated with higher CRP levels, and both malnutrition and inflammation were associated with dysfunctional neutrophils’ phagocytic activity, thus impairing immunity and favoring the maintenance of inflammation [32].

In uremic patients, AGEs are correlated with CRP and TNFα [15]. First of all, AGEs increase ROS production through the activation of NADPH oxidase and the interference with mitochondrial metabolism [33,34]. This process takes place both through AGE/RAGE interaction and with a receptor-independent mechanism [35]. In particular, AGEs seem to elicit an inflammatory response through the interaction with their receptor, RAGE [36,37]. In fact, RAGE activation translates into a signaling cascade that ends with the transcription of Nf-Kβ, enhancing the synthesis of pro-inflammatory cytokines, both locally and systemically [38,39]. RAGE expression by activated endothelium also promotes leukocyte recruitment, and AGEs delay spontaneous apoptosis of monocytes, consequently contributing to the maintenance of a pro-inflammatory status [40,41,42].

In a recent study conducted by Suliman et al. in patients with end-stage renal disease, plasma pentosidine (a subtype of AGE) was associated with inflammation and with the development of malnutrition [14]. Some uremic toxins (i.e., indole acetic acid, Indoxyl sulfate, *p*-cresyl sulfate) can increase ROS production, enhancing oxidative stress in CKD patients. This leads to proteins, lipids, and DNA modifications, many of which are composed of protein carbonyl compounds, which are precursors of pentosidine, which derives from protein and lipids oxidation and glycation [43,44,45]. Uremic patients, in fact, show a deficiency of glyoxalase I (GLO-I), failing to detoxify reactive carbonyl compounds, determining increased levels of AGEs in these patients [9].

Luketin M et al. described a negative correlation between AGEs, LDL-cholesterol, triglycerides, and BMI in hemodialysis patients and a positive association between AGEs and malnutrition [46]. sRAGE was of limited significance, instead, in identifying malnutrition in hemodialysis patients [47]. Markers of malnutrition are important determinants of AGEs accumulation in hemodialysis patients, and interventions devoted to improving nutritional status prevent a further rise of AGEs in this population [48,49]. In a prospective cohort study, Viramontes et al. observed that in dialysis patients who died during the observation, malnourished patients had increased levels of AGEs compared to well-nourished ones. Anyway, although a link between AGEs and malnutrition was evident, both were independent risk factors for mortality in this population [50].

Concerning the therapeutic approach against AGEs, in patients with diabetes, AGE formation can be prevented by hypoglycemic medicines, vitamins, and smoking cessation [51]. Moreover, statins and telmisartan could inhibit RAGE expression, whereas statins, ACE inhibitors, rosiglitazone, and vitamin D could increase RAGE levels [52,53]. In addition to traditional hypoglycemic and anti-hypertensive drugs, a number of biomolecules and phytochemicals (i.e., quercetin, sulforaphane, iridoids, curcumin) isolated from vegetables, legumes, fruits, or flavonoids are expected to become novel therapeutic agents. In fact, in cell-based and animal studies, it has been demonstrated that these compounds inhibit AGE formation, break preformed AGE, block AGE–RAGE axis, and stimulate glyoxalase [52,54,55,56,57,58].

Differently from previous studies, we did not find any significant difference in serum AGEs concentrations in well-nourished patients with respect to those that were at risk of malnutrition or malnourished. Instead, we observed an association between malnutrition and increased levels of sRAGE and esRAGE. The lack of a direct correlation between AGEs and malnutrition may depend on several discrepancies between our study and the previous ones. First of all, our study evaluated patients with advanced CKD but not yet in dialysis; therefore, we did not find such pronounced AGEs accumulation as it is described in dialyzed patients. Secondarily, eGFR, which is the main determinant of AGEs accumulation in CKD, was the same in the three groups of patients classified according to their nutritional status, and this may have nullified the possible effect of different degrees of renal dysfunction on AGEs accumulation. However, despite the fact that AGEs were not associated with malnutrition, we found that malnourished patients had higher levels of CRP that were directly associated with AGEs and AGEs/sRAGE ratio (Figure S2, Supplementary Materials). This suggests that in CKD patients’ inflammation may be amplified by and modulate AGEs/RAGEs interaction and production. In particular, this may be reflected by the fact that sRAGE levels (corrected for renal function) are directly related to inflammatory markers, such as CRP and TNFα. (Figure S1, Supplementary Materials). Therefore, although our study cannot be considered mechanistic, we described for the first time an intriguing relationship between systemic inflammation, AGEs/RAGEs interaction, and malnutrition in CKD patients.

We acknowledge that our study has some limitations. First of all, the cross-sectional design does not allow for attributing any causal relationship to the association between sRAGE isoforms levels and the onset of malnutrition in CKD patients. Secondly, our study is monocentric, and our population is relatively small. However, the monocentric nature of our study allowed us to reduce the possible sources of bias by using a highly standardized protocol for patients’ selection, biochemical analyses, and clinical observations. In particular, we applied strict inclusion and exclusion criteria that let us exclude patients that may have developed malnutrition because of specific clinical conditions.

A significant point of strength of our study is that the relationship between inflammation, AGEs, RAGEs, and malnutrition was thoroughly investigated. Furthermore, we studied in depth the association of AGEs and malnutrition by addressing not only AGEs but also RAGEs isoforms and the modulation of AGEs/RAGEs interaction played by inflammation. This is, to the best of our knowledge, the first time that a comprehensive evaluation of the association between the AGEs–RAGE system and malnutrition has been conducted in CKD patients not undergoing dialysis.

## 5. Conclusions

Our study shows that RAGEs levels are independently associated with malnutrition in patients with advanced CKD. Furthermore, we found that in these patients, inflammation can modulate AGEs/RAGEs interaction, indicating a possible correlation between inflammation, oxidative stress, and malnutrition. Whether these findings were confirmed, we could hypothesize that the modulation of AGEs/RAGEs interaction may contribute to preventing the development of malnutrition in this high-risk population.

## Figures and Tables

**Table 1:** General cohort characteristics.

Variables	Overall Cohort (*n* = 117)
*General characteristics*	
Age, (years)	80 ± 11
Males/Females, n (%)	82 (70)/35 (30)
Diabetes, n (%)	65 (56)
BMI, (kg/m^2^)	28 ± 5
MIS	
Not Malnourished, n (%)	37 (31)
At risk of malnutrition; n (%)	51 (43)
Malnourished, n (%)	30 (25)
eGFR, (mL/min/1.73 m^2^)	25 ± 11
Aetiology	
Nephroangiosclerosis, n (%)	44 (38)
Glomerulonephritis, n (%)	11 (9)
ADPKD, n (%)	2 (2)
Diabetic Nephropathy, n (%)	12 (10)
Other, n (%)	48 (41)
*Metabolic characteristics*	
Uric Acid, (mg/dL)	6.0 ± 1.5
HbA1c, (mmol/mol)	47 ± 11
Total Cholesterol, (mg/dL)	168 ± 37
HDL-Cholesterol, (mg/dL)	51 ± 15
LDL- Cholesterol, (mg/dL)	88 ± 32
Triglycerides, (mg/dL)	130 ± 54
Albumin, (g/dL)	4.0 ± 0.4
Prealbumin, (mg/dL)	28 ± 5
Proteinuria 24 h (g/24 h)	1.2 ± 1.6
*Inflammatory status and oxidative stress*	
CRP, (mg/dL)	0.4 ± 0.7
TNF alpha, (pg/mL)	15.3 ± 8.2
IL-6, (pg/mL)	4.2 ± 3.1
MCP-1, (pg/mL)	410 ± 176

Note: BMI: Body mass index; MIS: malnutrition-inflammation score; eGFR: estimated glomerular filtration rate; ADPKD: Autosomal Dominant Polycystic Kidney Disease; HbA1c: glycated hemoglobin; HDL: high density lipoprotein; LDL: low density lipoprotein; CRP: c-reactive protein; TNFα: Tumor necrosis factor alpha; IL-6, interleukin 6; MCP-1: Monocyte chemotactic protein-1. Data are expressed as mean with standard deviation or number and percentages.

**Table 2:** Comparison of general characteristics and nutritional parameters between not malnourished, at risk of malnutrition and malnourished patients.

Variables [n.r.]	Not Malnourished (*n* = 37)	Risk of Malnutrition (*n* = 51)	Malnourished (*n* = 29)	*p* *
Males, n (%)	30 (81)	35 (69)	18 (62)	0.16
Diabetes, n (%)	17 (46)	32 (63)	16 (55)	0.29
Age (years)	72 ± 17	77 ± 8	82 ± 5	**0.002**
BMI, (kg/m^2^)	28 ± 5	28.1 ± 5	26.7 ± 4.7	0.22
eGFR, (mL/min/1.73 m^2^)	27 ± 12	25 ± 11	21 ± 10	0.11
*Body composition and metabolic characteristics*				
MIS	3 [3–4]	4 [3–5]	10 [8–13]	**<0.000**
nPCR (g/kg/24 h)	0.8 ± 0.3	0.8 ± 0.2	0.65 ± 0.2	**0.01**
Urinary creatinine (mg/24 h) [1000–2300]	1064 ± 341	1128 ± 1303	775 ± 261	0.23
Calories (Kcal/kg)	22 ± 9	22 ± 6	21 ± 6	0.8
HbA1c, (mmol/mol) [20–42]	45 ± 8	50 ± 13	45 ± 10	0.11
Total Cholesterol, (mg/dL) [<190]	175 ± 37	166 ± 40	163 ± 31	0.76
HDL-Cholesterol, (mg/dL) [m > 38, f > 42]	58 ± 23	49 ± 15	53 ± 15	0.22
LDL-Cholesterol, (mg/dL) [<115]	92 ± 26	90 ± 36	84 ± 23	0.4
Triglycerides, (mg/dL) [<150]	127 ± 54	131 ± 49	128 ± 61	0.8
Albumin, (g/dL) [3.4–4.8]	4.2 ± 0.3	4.1 ± 0.4	3.8 ± 0.2	**0.002**
Prealbumin, (mg/dL) [23–42]	32 ± 5	27 ± 5	27 ± 6	**<0.0001**

Note: n.r.: normal range; BMI: Body mass index; eGFR: estimated glomerular filtration rate; MIS: malnutrition inflammation score; nPCR: normalized protein catabolic rate HbA1c: glycated hemoglobin; HDL: high density lipoprotein; LDL: low density lipoprotein. Data are expressed as mean with standard deviation or number and percentages. * *p* values are intended for trend and values less than 0.05 are indicated in bold.

**Table 3:** Comparison of inflammatory markers between not malnourished, at risk of malnutrition and malnourished patients.

Variables [n.r.]	Not Malnourished (*n* = 37)	Risk of Malnutrition (*n* = 51)	Malnourished (*n* = 29)	*p* *
CRP, (mg/dL) [<0.5]	0.2 ± 0.1	0.6 ± 0.7	0.6 ± 1	**0.02**
TNFα, (pg/mL) [14.6]	12 ± 6	16 ± 8	15 ± 9	**0.029**
IL-6, (pg/mL) [1.45]	6 ± 16	4 ± 3	4 ± 3	0.76
MCP-1, (pg/mL) [31.2–2000]	422 ± 158	437 ± 142	410 ± 176	0.49

Note: n.r.: normal range; CRP: c-reactive protein; TNFα: Tumor necrosis factor alpha; IL-6: interleukin 6, MCP-1: Monocyte chemotactic protein-1. Data are expressed as mean with standard. * *p* values are intended for trend and values less than 0.05 are indicated in bold.

**Table 4:** Concentration of AGEs and sRAGE isoforms in not malnourished, at risk of malnutrition and malnourished CKD patients.

Variables	Not Malnourished (*n* = 37)	Risk of Malnutrition (*n* = 51)	Malnourished (*n* = 29)	*p*
AGEs (arbitrary unit)	2960 ± 854	3031 ± 779	3079 ± 780	0.79
sRAGE (pg/mL)	2314 ± 1115	2158 ± 1236	2813 ± 1477	**0.035**
esRAGE (pg/mL)	545 [380–730]	476 [355–680]	648 [408–1049]	**0.033**
cRAGE (pg/mL)	1704 ± 844	1558 ± 929	1996 ± 1049	0.07
AGEs/sRAGE (arbitrary unit)	1.6 ± 1	1.8 ± 0.9	1.5 ± 0.97	0.17
cRAGE/esRAGE	2.96 ± 0.9	2.8 ± 0.9	2.7 ± 1.1	0.61

Note: AGEs: Advanced Glycation End products; sRAGE: soluble receptor for AGE; esRAGE: endogenous secretory receptor for AGE; cRAGE: cleaved receptor for AGE; CKD: chronic kidney disease. Data are expressed as mean with standard deviation. *p* values less than 0.05 are indicated in bold.

**Table 5:** Multivariable logistic regression model evaluating the relationship between AGEs, sRAGE and malnutrition development.

Nutritional Status	Variables	Odds Ratio	95% Confidence Interval	*p*
*Not Malnourished*	AGEs (arbitrary unit)	1.5	(0.5–2.6)	0.8
	sRAGE (arbitrary unit)	0.41	(0.29–0.86)	**0.035**
	CRP (mg/dL)	0.08	(0.01–0.12)	**0.001**
	Age (years)	0.87	(0.81–0.94)	**0.001**
	Sex (M)	2.68	(0.76–9.36)	0.126
	Sex (F)	0.37	(0.1–1.31)	
*At risk for Malnutrition*	AGEs (arbitrary unit)	1.3	(0.81–1.95)	0.926
	sRAGE (arbitrary unit)	1.4	(0.52–2.62)	0.95
	CRP (mg/dL)	9.2	(5.8–14.6)	**0.001**
	Age (years)	1.04	(0.99–1.09)	0.106
	Sex (M)	0.56	(0.48–3.73)	0.229
	Sex (F)	1.85	(0.26–2.1)	
*Malnourished*	AGEs (arbitrary unit)	0.56	(0.46–1.53)	0.97
	sRAGE (arbitrary unit)	2.22	(1.16–3.44)	**0.035**
	CRP (mg/dL)	12.13	(7.4–20.5)	**0.001**
	Age (years)	1.41	(1.05–1.23)	**0.001**
	Sex (M)	0.37	(0.1–1.31)	0.126
	Sex (F)	2.7	(0.76–9.34)	
*Not Malnourished*	AGEs (arbitrary unit)	1.5	(0.5–2.6)	0.8
	esRAGE (arbitrary unit)	0.75	(0.42–1.02)	0.055
	CRP (mg/dL)	0.008	(0.00–0.14)	**0.001**
	Age (years)	0.87	(0.8–0.94)	**0.001**
	Sex (M)	2.5	(0.7–8.9)	0.160
	Sex (F)	0.4	(0.11–1.44)	
*At risk for Malnutrition*	AGEs (arbitrary unit)	1.23	(0.87–1.74)	0.931
	esRAGE (arbitrary unit)	1.32	(0.45–3.1)	0.95
	CRP (mg/dL)	9.5	(5.6–15.9)	**0.002**
	Age (years)	1.04	(0.99–1.09)	0.107
	Sex (M)	1.3	(0.46–3.64)	0.62
	Sex (F)	0.77	(0.27–2.17)	
*Malnourished*	AGEs (arbitrary unit)	0.8	(0.7–1.3)	0.98
	esRAGE (arbitrary unit)	1.33	(0.98–2.38)	0.055
	CRP (mg/dL)	11.9	(6.8–20.6)	**0.001**
	Age (years)	1.14	(1.05–1.24)	**0.001**
	Sex (M)	0.4	(0.11–1.43)	0.16
	Sex (F)	2.5	(0.69–8.92)	

Note: AGEs: Advanced Glycation End products; sRAGE: soluble receptor for AGE; CRP: C reactive protein; M: male; F: female. Each model was built evaluating variable that showed interaction with malnutrition development in previous analyses. Not malnourished patients (MIS score < 4), at risk for malnutrition (MIS score 4–7), and overtly malnourished patients (MIS ≥ 8). *p* values less than 0.05 are indicated in bold.

## Data Availability

The dataset analyzed for this study can be found in the OSF repository at https://osf.io/m5k74/files/ (accessed on 29 May 2022).

## Supplementary Materials

The following supporting information can be downloaded at: https://www.mdpi.com/article/10.3390/antiox11071253/s1, Figure S1: linear regression analyses evaluating the association between sRAGE and esRAGEwith markers of inflammation, weighted for eGFR. Figure S2: linear regression analyses evaluating the association between AGE and AGEs/sRAGE ratio with markers of inflammation, weighted for eGFR. Table S1: linear correlation comparison between body composition parameters and AGEs and RAGEs isoforms levels. Statistical significance is reported in the table. table. Table S2: Interaction analysis using logistic regression model, between AGEs, sRAGE and esRAGE, biochemical and metabolic parameters, and malnutrition development.
