# Supplementary material for: In Patients with Chronic Kidney Disease Advanced Glycation End-Products Receptors Isoforms (sRAGE and esRAGE) Are Associated with Malnutrition
Source: Antioxidants (Basel). 2022 Jun 25;11(7):1253. doi: 10.3390/antiox11071253 (PMC9312066; doi:10.3390/antiox11071253)
Supplement: Supplementary file 1 [file antioxidants-11-01253-s001.zip › antioxidants-1774441-supplementary.pdf]

Supplementary materials

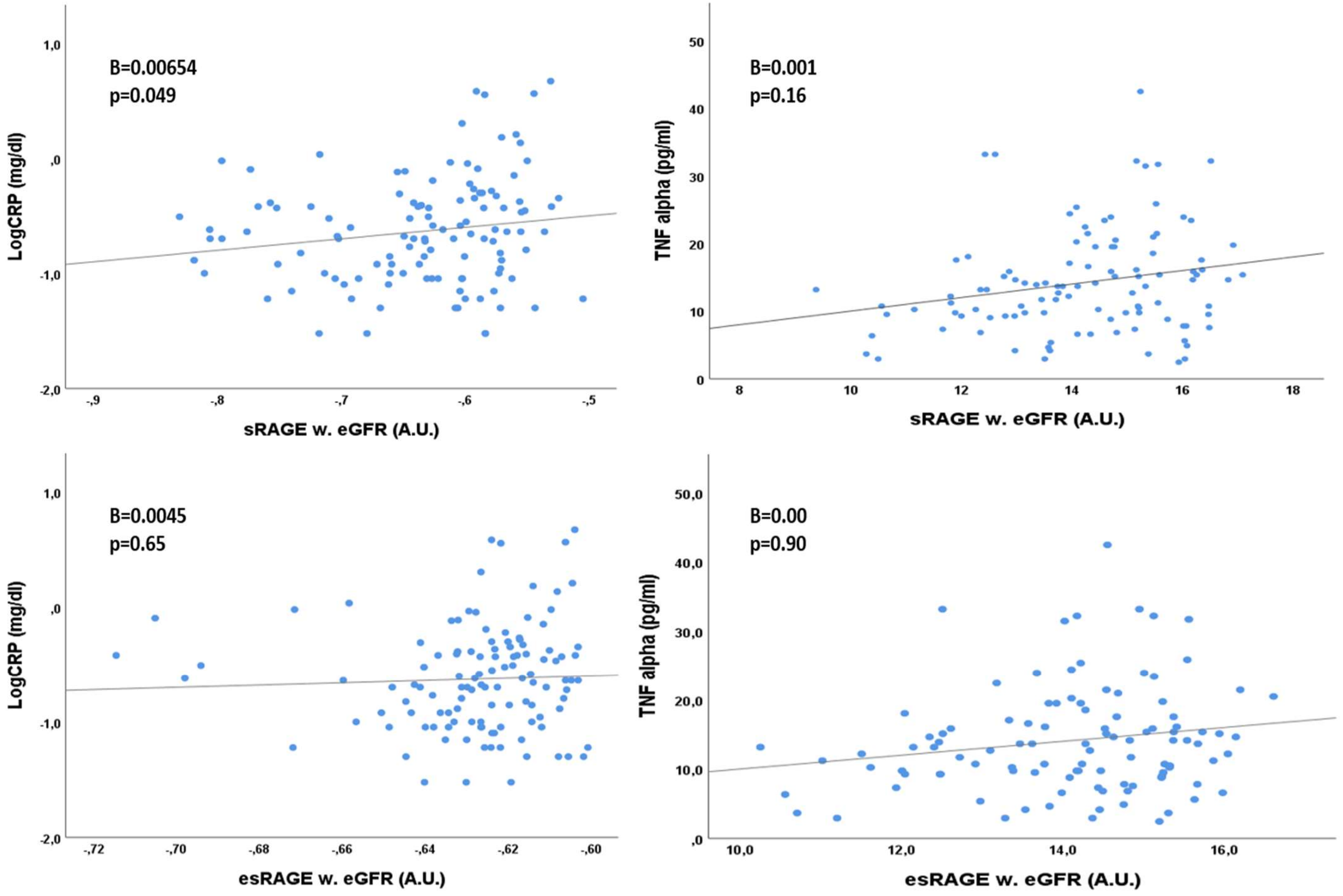

Figure S1: linear regression analyses evaluating the association between sRAGE and esRAGE with markers of inflammation weighted for eGFR.

Note: CRP: c-reactive protein; TNFα: tumor necrosis factor α; sRAGE: sRAGE, soluble receptor for AGE; esRAGE: endogenous secretory receptor for AGE; eGFR: estimated glomerular filtration rate.

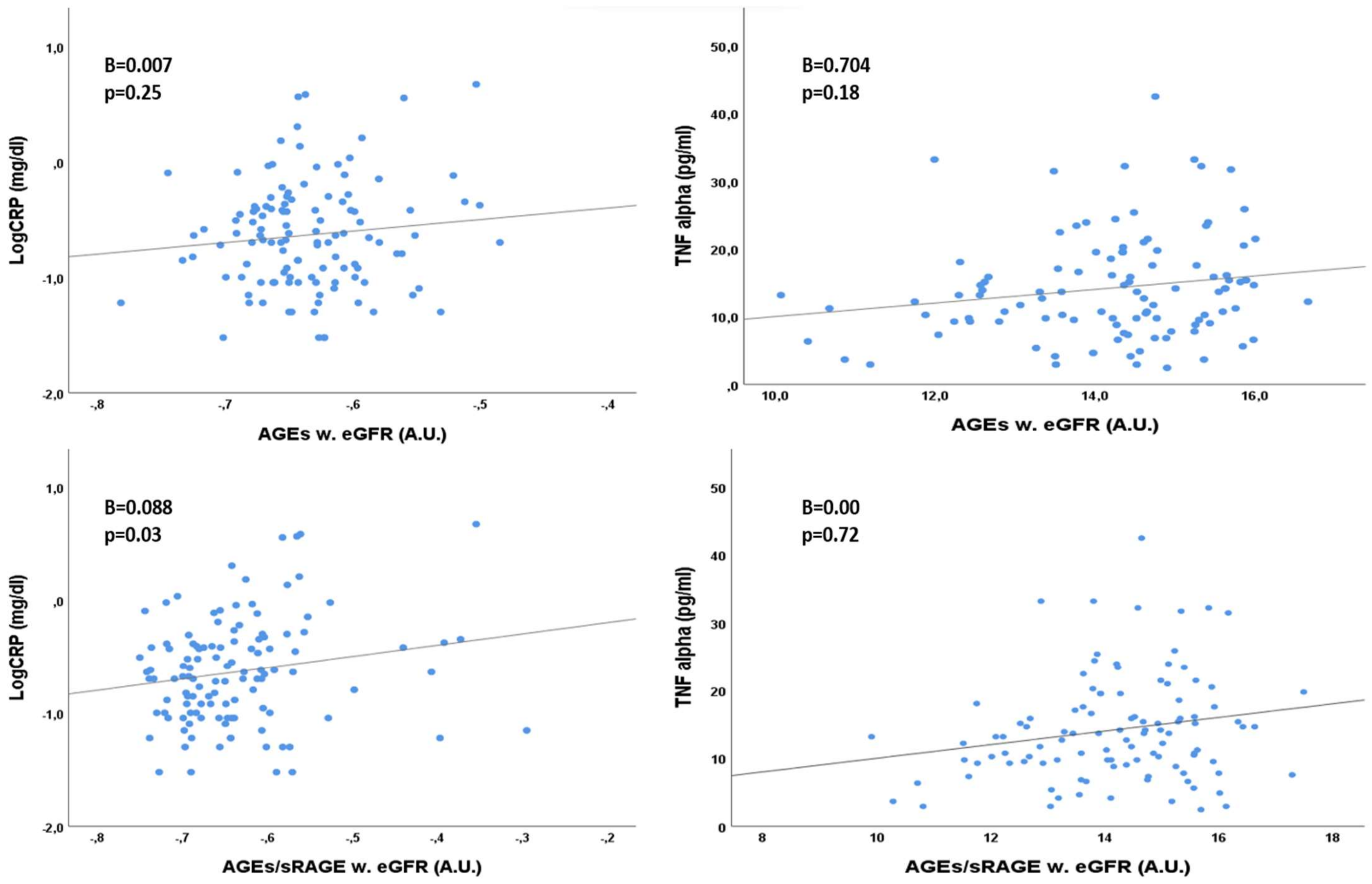

Figure S2: linear regression analyses evaluating the association between AGE and AGEs/sRAGE ratio with markers of inflammation, weighted for eGFR

Note: CRP: c-reactive protein; TNFα: tumor necrosis factor α; AGEs: Advanced Glycation End products; sRAGE, soluble receptor for AGE; eGFR: estimated glomerular filtration rate.

Table S1: linear correlation comparison between body composition parameters and AGEs and RAGEs isoforms levels.

Variables	OH		LTI		FTI		BCM	
	**Pearson coefficient**	**p**	**Pearson coefficient**	**p**	**Pearson coefficient**	**p**	**Pearson coefficient**	**p**
AGEs (arbitrary unit)	0.179	0.095	-0.052	0.630	-0.114	0.292	-0.042	0.695
sRAGE (pg/mL)	0.173	0.106	-0.076	0.483	-0.087	0.751	-0.066	0.539
esRAGE (pg/ml)	**0.264**	**0.013**	-0.047	0.662	-0.056	0.422	-0.039	0.717
cRAGE (pg/ml)	0.107	0.319	-0.081	0.452	-0.034	0.751	-0.072	0.504
AGEs/sRAGE (arbitrary unit)	-0.073	0.502	0.097	0.367	-0.030	0.780	0.088	0.417

Note: AGEs: Advanced Glycation End products; sRAGE. soluble receptor for AGE; esRAGE: endogenous secretory receptor for AGE; cRAGE: cleaved receptor for AGE; CKD. chronic kidney disease. Data are expressed as mean with standard deviation. P values less than 0.05 are indicated in bold.

Table S2: Interaction analysis using logistic regression model, between AGEs, sRAGE and esRAGE, biochemical and metabolic parameters, and malnutrition development.

Dependent variable	Control variable	Variables	B value	p
Malnutrition development	*AGEs*	Age (years)	-1.026	0.09
		BMI (Kg/m^2)	-1.026	0.663
		eGFR (ml/min/m^2)	-1.026	0.548
		Albumin (g/dl)	-1.065	0.384
		CRP (mg/dl)	-1.076	0.257
		TNFα (pg/ml)	-1.074	0.503
		MCP-1 (pg/ml)	-0.901	0.633
		Diabetes	-1.076	0.996
		Sex	-1.076	0.243
	sRAGE	Age (years)	-1.076	**0.003**
		BMI (Kg/m^2)	-1.076	0.107
		eGFR (ml/min/m^2)	-1.076	0.781
		Albumin (g/dl)	-1.065	0.104
		CRP (mg/dl)	-1.076	**0.033**
		TNFα (pg/ml)	-1.074	0.246
		MCP-1 (pg/ml)	-0.901	0.206
		Diabetes	-1.076	0.421

		Sex	-1.076	**0.017**
	esRAGE	Age (years)	-1.110	**0.002**
		BMI (Kg/m^2)	-1.110	**0.032**
		eGFR (ml/min/m^2)	-1.110	0.350
		Albumin (g/dl)	-1.099	**0.031**
		CRP (mg/dl)	-1.110	**0.023**
		TNFα (pg/ml)	-1.111	0.259
		MCP-1 (pg/ml)	-0.901	0.259
		Diabetes	-1.110	0.383
		Sex	-1.110	**0.004**

Note: BMI: Body mass index; eGFR: estimated glomerular filtration rate; nPCR: normalized protein catabolic rate; CRP: c-reactive protein; TNFα: Tumor necrosis factor alpha; IL-6: interleukin 6, MCP-1: Monocyte chemotactic protein-1. The model was built evaluating the interaction between AGEs, sRAGE, esRAGE and variables showed in the table for malnutrition development (malnutrition: dependent variable – Control variable * variable as independent variable). P values less than 0.05 are indicated in bold.
